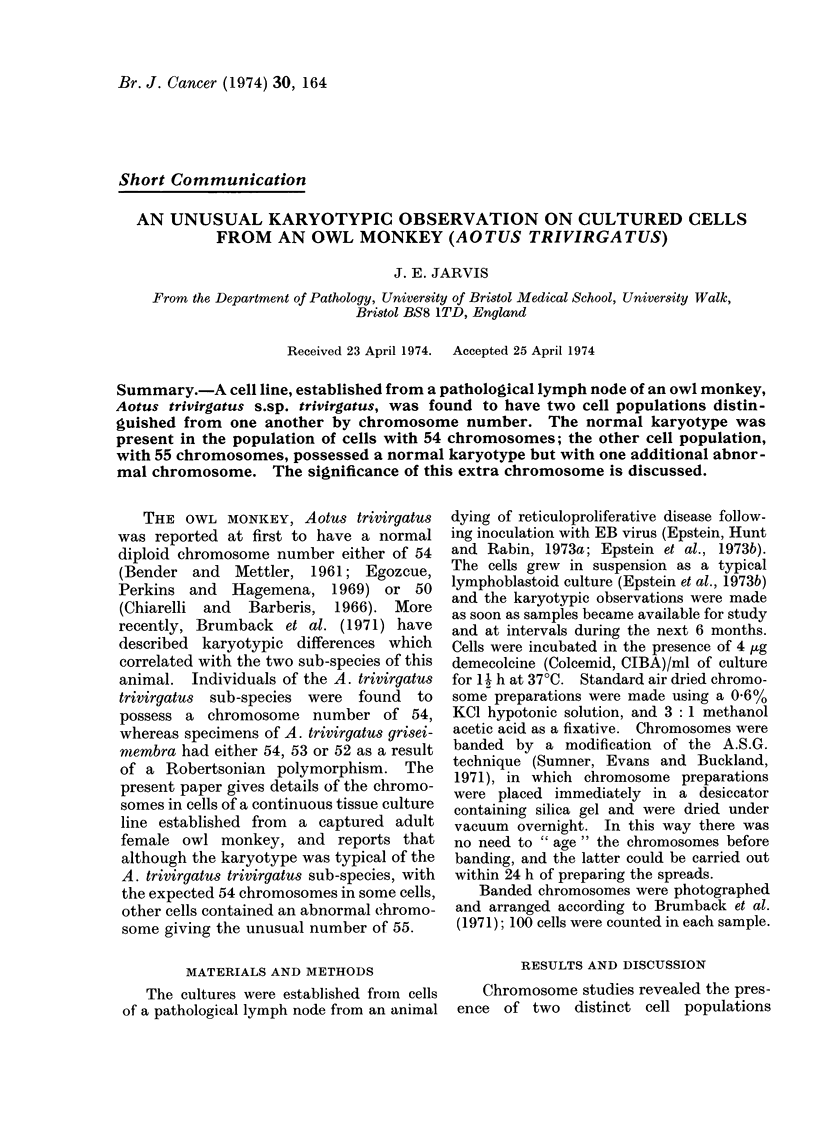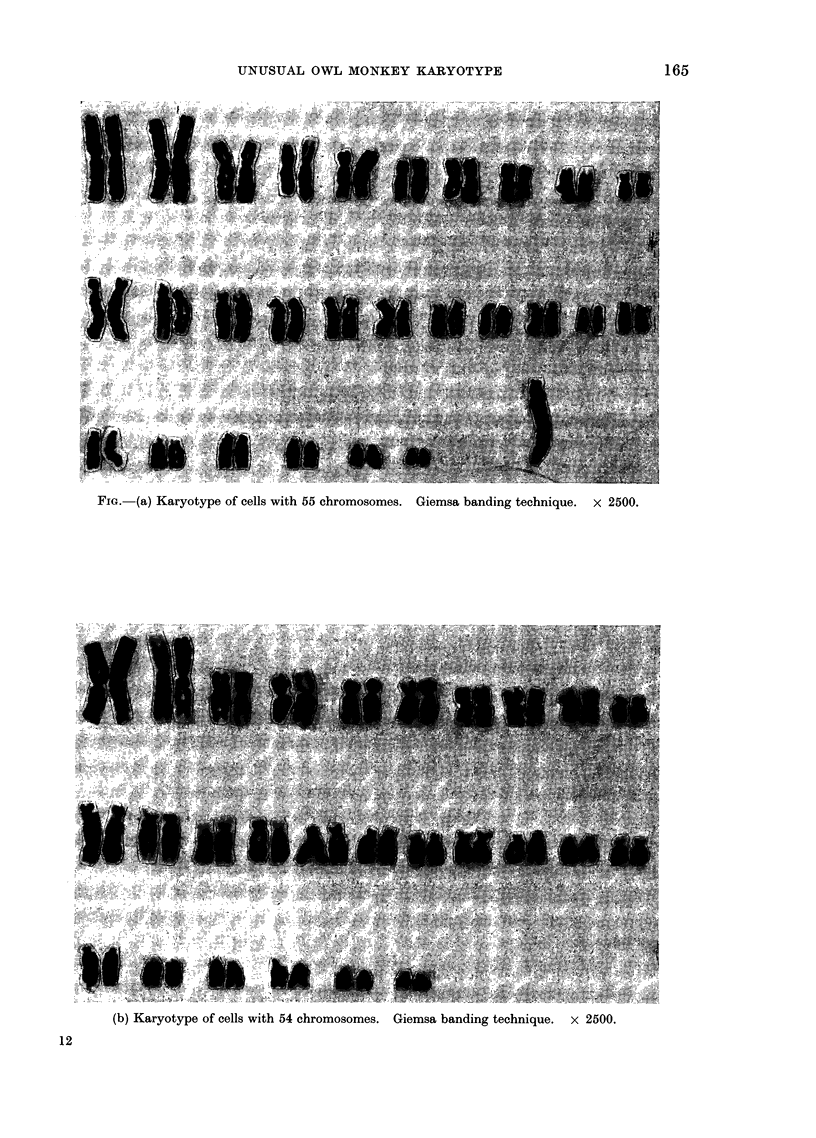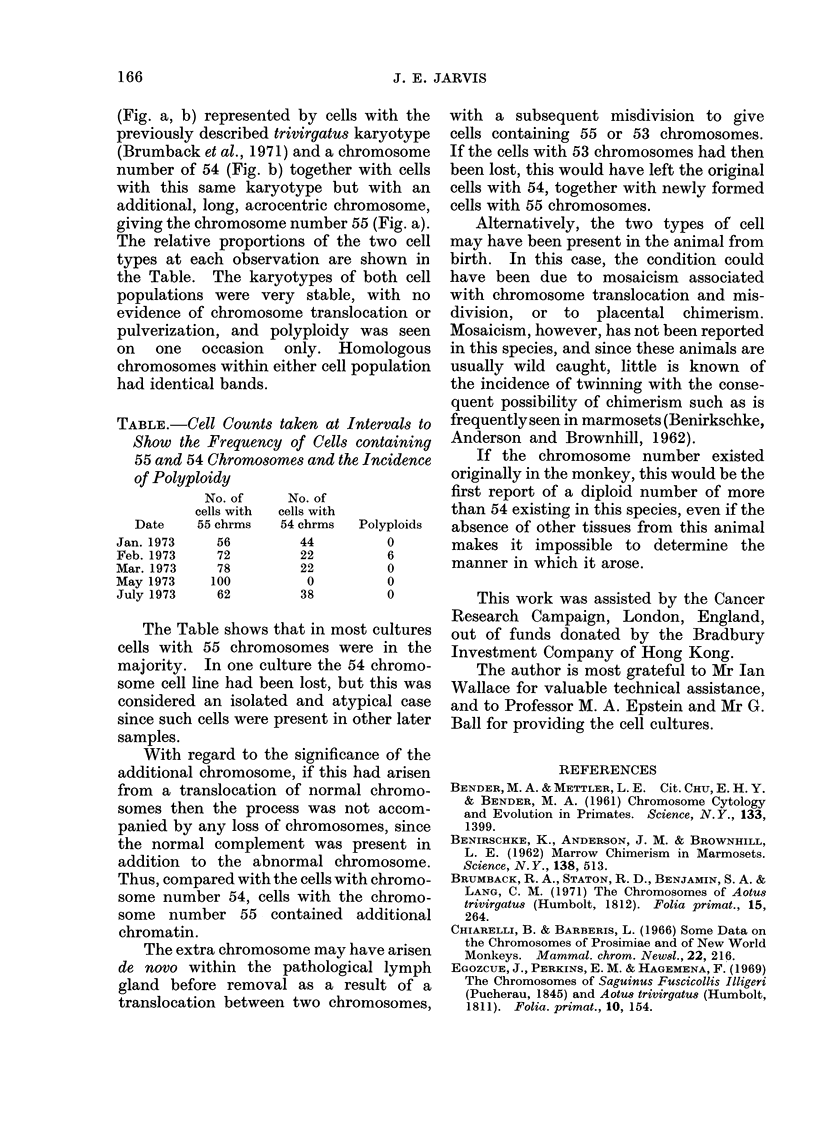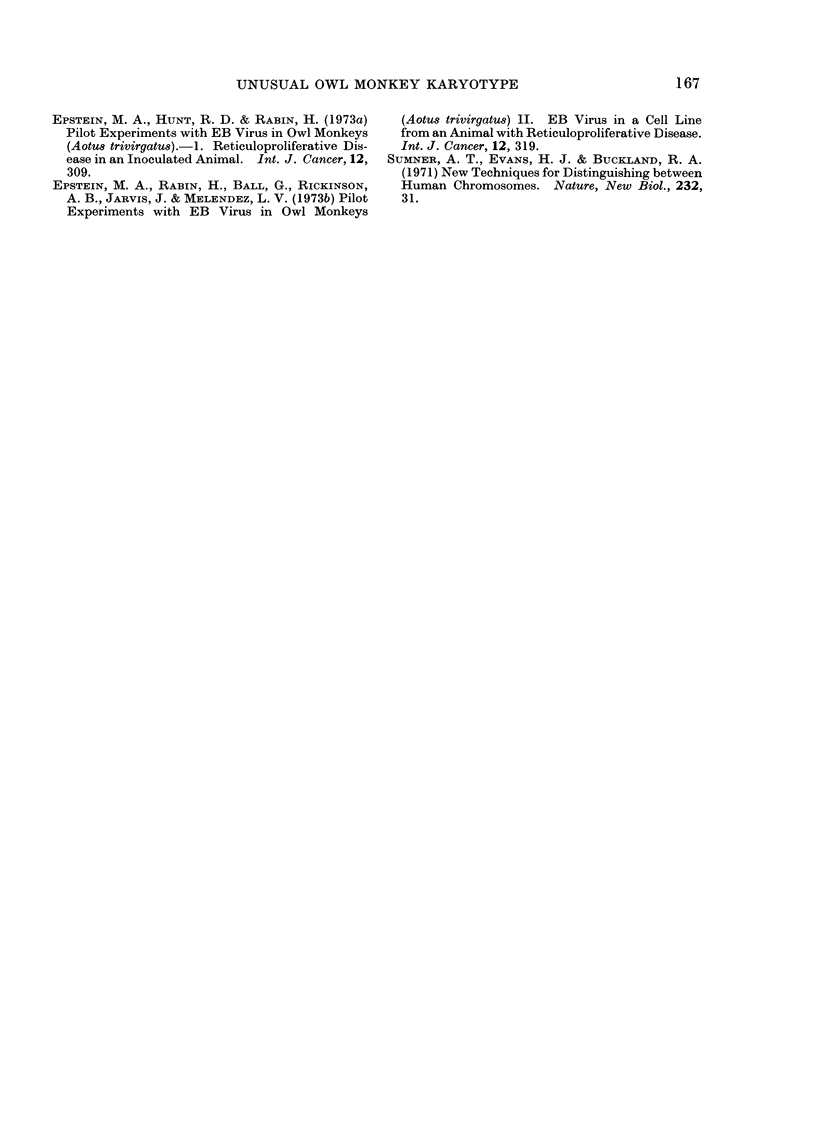# An Unusual Karyotypic Observation on Cultured Cells from an Owl Monkey (Aotus Trivirgatus)

**DOI:** 10.1038/bjc.1974.128

**Published:** 1974-08

**Authors:** J. E. Jarvis

## Abstract

**Images:**


*Br. J. Cancer* (1974) **30**, 164

***Short Communication***

### AN UNUSUAL KARYOTYPIC OBSERVATION ON CULTURED CELLS FROM AN OWL MONKEY (*AOTUS TRIVIRGATUS*)

J. E. JARVIS

*From the Department of Pathology, University of Bristol Medical School, University Walk, Bristol BS8 1TD, England*

Received 23 April 1974. Accepted 25 April 1974

**Summary.—A cell line, established from a pathological lymph node of an owl monkey, *Aotus trivirgatus* s.sp. *trivirgatus*, was found to have two cell populations distinguished from one another by chromosome number. The normal karyotype was present in the population of cells with 54 chromosomes; the other cell population, with 55 chromosomes, possessed a normal karyotype but with one additional abnormal chromosome. The significance of this extra chromosome is discussed.**

THE OWL MONKEY, *Aotus trivirgatus* was reported at first to have a normal diploid chromosome number either of 54 (Bender and Mettler, 1961; Egozcue, Perkins and Hagemena, 1969) or 50 (Chiarelli and Barberis, 1966). More recently, Brumback *et al.* (1971) have described karyotypic differences which correlated with the two sub-species of this animal. Individuals of the *A. trivirgatus trivirgatus* sub-species were found to possess a chromosome number of 54, whereas specimens of *A. trivirgatus griseimembra* had either 54, 53 or 52 as a result of a Robertsonian polymorphism. The present paper gives details of the chromosomes in cells of a continuous tissue culture line established from a captured adult female owl monkey, and reports that although the karyotype was typical of the *A. trivirgatus trivirgatus* sub-species, with the expected 54 chromosomes in some cells, other cells contained an abnormal chromosome giving the unusual number of 55.

### MATERIALS AND METHODS

The cultures were established from cells of a pathological lymph node from an animal dying of reticuloproliferative disease following inoculation with EB virus (Epstein, Hunt and Rabin, 1973*a*; Epstein *et al.*, 1973*b*). The cells grew in suspension as a typical lymphoblastoid culture (Epstein *et al.*, 1973*b*) and the karyotypic observations were made as soon as samples became available for study and at intervals during the next 6 months. Cells were incubated in the presence of 4 $\mu$g demecolcine (Colcemid, CIBA)/ml of culture for $1\frac{1}{2}$ h at 37°C. Standard air dried chromosome preparations were made using a 0·6% KCl hypotonic solution, and 3 : 1 methanol acetic acid as a fixative. Chromosomes were banded by a modification of the A.S.G. technique (Sumner, Evans and Buckland, 1971), in which chromosome preparations were placed immediately in a desiccator containing silica gel and were dried under vacuum overnight. In this way there was no need to " age " the chromosomes before banding, and the latter could be carried out within 24 h of preparing the spreads.

Banded chromosomes were photographed and arranged according to Brumback *et al.* (1971); 100 cells were counted in each sample.

### RESULTS AND DISCUSSION

Chromosome studies revealed the presence of two distinct cell populations

FIG.—(a) Karyotype of cells with 55 chromosomes. Giemsa banding technique. × 2500.

(b) Karyotype of cells with 54 chromosomes. Giemsa banding technique. × 2500.

(Fig. a, b) represented by cells with the previously described *trivirgatus* karyotype (Brumback *et al.*, 1971) and a chromosome number of 54 (Fig. b) together with cells with this same karyotype but with an additional, long, acrocentric chromosome, giving the chromosome number 55 (Fig. a). The relative proportions of the two cell types at each observation are shown in the Table. The karyotypes of both cell populations were very stable, with no evidence of chromosome translocation or pulverization, and polyploidy was seen on one occasion only. Homologous chromosomes within either cell population had identical bands.

TABLE.—*Cell Counts taken at Intervals to Show the Frequency of Cells containing 55 and 54 Chromosomes and the Incidence of Polyploidy*

| Date | No. of cells with 55 chrms | No. of cells with 54 chrms | Polyploids |
|---|---|---|---|
| Jan. 1973 | 56 | 44 | 0 |
| Feb. 1973 | 72 | 22 | 6 |
| Mar. 1973 | 78 | 22 | 0 |
| May 1973 | 100 | 0 | 0 |
| July 1973 | 62 | 38 | 0 |

The Table shows that in most cultures cells with 55 chromosomes were in the majority. In one culture the 54 chromosome cell line had been lost, but this was considered an isolated and atypical case since such cells were present in other later samples.

With regard to the significance of the additional chromosome, if this had arisen from a translocation of normal chromosomes then the process was not accompanied by any loss of chromosomes, since the normal complement was present in addition to the abnormal chromosome. Thus, compared with the cells with chromosome number 54, cells with the chromosome number 55 contained additional chromatin.

The extra chromosome may have arisen *de novo* within the pathological lymph gland before removal as a result of a translocation between two chromosomes, with a subsequent misdivision to give cells containing 55 or 53 chromosomes. If the cells with 53 chromosomes had then been lost, this would have left the original cells with 54, together with newly formed cells with 55 chromosomes.

Alternatively, the two types of cell may have been present in the animal from birth. In this case, the condition could have been due to mosaicism associated with chromosome translocation and misdivision, or to placental chimerism. Mosaicism, however, has not been reported in this species, and since these animals are usually wild caught, little is known of the incidence of twinning with the consequent possibility of chimerism such as is frequently seen in marmosets (Benirkschke, Anderson and Brownhill, 1962).

If the chromosome number existed originally in the monkey, this would be the first report of a diploid number of more than 54 existing in this species, even if the absence of other tissues from this animal makes it impossible to determine the manner in which it arose.

This work was assisted by the Cancer Research Campaign, London, England, out of funds donated by the Bradbury Investment Company of Hong Kong.

The author is most grateful to Mr Ian Wallace for valuable technical assistance, and to Professor M. A. Epstein and Mr G. Ball for providing the cell cultures.

### REFERENCES

BENDER, M. A. & METTLER, L. E. Cit. CHU, E. H. Y. & BENDER, M. A. (1961) Chromosome Cytology and Evolution in Primates. *Science, N.Y.*, **133**, 1399.

BENIRSCHKE, K., ANDERSON, J. M. & BROWNHILL, L. E. (1962) Marrow Chimerism in Marmosets. *Science, N.Y.*, **138**, 513.

BRUMBACK, R. A., STATON, R. D., BENJAMIN, S. A. & LANG, C. M. (1971) The Chromosomes of *Aotus trivirgatus* (Humbolt, 1812). *Folia primat.*, **15**, 264.

CHIARELLI, B. & BARBERIS, L. (1966) Some Data on the Chromosomes of Prosimiae and of New World Monkeys. *Mammal. chrom. Newsl.*, **22**, 216.

EGOZCUE, J., PERKINS, E. M. & HAGEMENA, F. (1969) The Chromosomes of *Saguinus Fuscicollis Illigeri* (Pucherau, 1845) and *Aotus trivirgatus* (Humbolt, 1811). *Folia. primat.*, **10**, 154.

Epstein, M. A., Hunt, R. D. & Rabin, H. (1973*a*) Pilot Experiments with EB Virus in Owl Monkeys (*Aotus trivirgatus*).—1. Reticuloproliferative Disease in an Inoculated Animal. *Int. J. Cancer*, **12**, 309.

Epstein, M. A., Rabin, H., Ball, G., Rickinson, A. B., Jarvis, J. & Melendez, L. V. (1973*b*) Pilot Experiments with EB Virus in Owl Monkeys (*Aotus trivirgatus*) II. EB Virus in a Cell Line from an Animal with Reticuloproliferative Disease. *Int. J. Cancer*, **12**, 319.

Sumner, A. T., Evans, H. J. & Buckland, R. A. (1971) New Techniques for Distinguishing between Human Chromosomes. *Nature, New Biol.*, **232**, 31.